# Role of *Chlamydia trachomatis* in Miscarriage

**DOI:** 10.3201/eid1709.100865

**Published:** 2011-09

**Authors:** David Baud, Genevieve Goy, Katia Jaton, Maria-Chiara Osterheld, Serafin Blumer, Nicole Borel, Yvan Vial, Patrick Hohlfeld, Andreas Pospischil, Gilbert Greub

**Affiliations:** Author affiliations: University Hospital of Lausanne, Lausanne, Switzerland (D. Baud, G. Goy, K. Jaton, M.-C. Osterheld, Y. Vial, P. Hohlfeld, G. Greub);; University of Zürich, Zürich, Switzerland (S. Blumer, N. Borel, A. Pospischil)

**Keywords:** Chlamydia trachomatis, abortion, adverse pregnancy outcome, placental infection, sexually transmitted disease, miscarriage, bacteria, research

## Abstract

To determine the role of *Chlamydia trachomatis* in miscarriage, we prospectively collected serum, cervicovaginal swab specimens, and placental samples from 386 women with and without miscarriage. Prevalence of immunoglobulin G against *C. trachomatis* was higher in the miscarriage group than in the control group (15.2% vs. 7.3%; p = 0.018). Association between *C. trachomatis*–positive serologic results and miscarriage remained significant after adjustment for age, origin, education, and number of sex partners (odds ratio 2.3, 95% confidence interval 1.1–4.9). *C. trachomatis* DNA was more frequently amplified from products of conception or placenta from women who had a miscarriage (4%) than from controls (0.7%; p = 0.026). Immunohistochemical analysis confirmed *C. trachomatis* in placenta from 5 of 7 patients with positive PCR results, whereas results of immunohistochemical analysis were negative in placenta samples from all 8 negative controls tested. Associations between miscarriage and serologic/molecular evidence of *C. trachomatis* infection support its role in miscarriage.

## Abstract 2

TOC Summary: Women experiencing miscarriage should be screened for *C. trachomatis*.

The incidence of *Chlamydia trachomatis* infection has dramatically increased during the past 10 years (*1*). Mostly asymptomatic, untreated *C. trachomatis* infections are responsible for a large proportion of salpingitis, pelvic inflammatory disease, ectopic pregnancy, and infertility in women. *C. trachomatis* is a recognized agent of preterm labor and premature rupture of membranes (*2**,**3*). However, its role in miscarriage is unclear (*2**,**3*).

*C. trachomatis* has been isolated or detected in cervical smear, urine (*4**–**6*), or products of conception (*7**,**8*). Nevertheless, none of these studies demonstrated association between isolation of *C. trachomatis* and miscarriage. However, culturing *C. trachomatis* is technically difficult, given its strict intracellular life cycle. Even with molecular approaches, detecting *C. trachomatis* can be difficult because of PCR inhibitors or low number of copies often present in the lesions (*4**–**7*). Moreover, infection could be localized at deeper sites not amenable to sampling (*9*).

Several studies have reported a higher prevalence of *C. trachomatis* antibodies in spontaneous (*10**,**11*) or recurrent (*2**,**9**,**11**,**12*) miscarriages. The inability to detect immunoglobulin (Ig) M or to isolate *C. trachomatis* from any of these seropositive patients might suggest that *Chlamydia* spp. are not directly associated with miscarriage (*9**,**12*). Other seroepidemiologic studies have failed to find any correlation between *C. trachomatis* and spontaneous (*13**–**16*) or recurrent miscarriage (*17**,**18*).

The main purpose of this study was to investigate whether *C. trachomatis* is associated with miscarriage. We used molecular, serologic, and immunohistochemical approaches to compare evidence of present and past *C. trachomatis* infection in women with or without miscarriage.

## Materials and Methods

During November 2006–June 2009, a total of 386 women were prospectively enrolled at the obstetric department of the University Hospital of Lausanne (Lausanne, Switzerland). The miscarriage group comprised 125 women consulting at the emergency gynecology ward for an acute miscarriage. The control group comprised 261 women attending the labor ward with an uneventful pregnancy and without any history of miscarriage, stillbirth, or preterm labor. All women gave written consent, and the local ethical committee approved the study.

We collected demographic and obstetric data prospectively. Placenta (or products of conception in cases of miscarriage), cervicovaginal swab specimens, and serum were sampled at the time of labor and of miscarriage.

All serum samples were tested for IgG and IgA against *C. trachomatis* with the Ridascreen *Chlamydia* IgG/IgA Kit (R-biopharm, Darmstadt, Germany) according to the manufacturer’s instructions and by using Dynex DSX (Magellan Biosciences, Chantilly, VA, USA). Cervicovaginal swabs and placenta were extracted by using QIAamp DNA Mini kit (QIAGEN, Hilden, Germany). Samples were screened for *C. trachomatis* DNA by using a TaqMan real-time PCR specific for the cryptic plasmid of *C. trachomatis*, as described (*19*). A PCR inhibition control was used to verify that absence of amplification was not caused by PCR inhibitors. Only 1 of the 386 PCR inhibition controls was negative. This sample was thus retested at a 1:10 dilution.

Hematoxylin and eosin–stained histologic sections of all placentas were investigated for deciduitis, vasculitis, endometritis, or chorioamnionitis. Histologic samples were read blindly by a pedopathologist (M.-C.O.). Samples positive for *C. trachomatis* by real-time PCR were tested by immunohistochemical analysis (IHC). Presence of *C. trachomatis* on histologic sections was assessed by using a specific mouse monoclonal antibody, as described (*20*). To test the placental specimens, we used a commercial *Chlamydiaceae* family–specific monoclonal antibody directed against the chlamydial lipopolysaccharide (clone AC1-P; Progen, Heidelberg, Germany) at a dilution of 1:200. Detection was performed with the Dako ChemMate detection Kit (Dako, Glostrup, Denmark) according to the manufacturer’s instructions. Antigen retrieval was performed by 10-min enzyme digestion (proteinase K; Dako). Immersion of the slides in peroxidase-blocking solution for 5 min at room temperature resulted in blocking of endogenous peroxidase activity. Specimens were incubated with primary antibody for 1 h. Sections were incubated for 10 min at room temperature with the link-antibody (Dako), followed by 10 min incubation with horseradish peroxidase (Dako) and finally developed in 3-amino, 9-ethyl-carbazole substrate solution for 10 min at room temperature and counterstained with hematoxylin. Using the antibody diluent instead of the primary antibody, we performed a negative control of each section. Moreover, 8 placentas from *C. trachomatis* PCR-negative patients were randomly selected as negative controls. IHC was blindly read by 2 pathologists with experience in chlamydial IHC (S.B., N.B.) and confirmed by a pedopathologist (M.-C.O.).

We compared demographic data and risk factors of patients with and without miscarriage or *C. trachomatis* infection by the Pearson χ^2^ test (or the Fisher exact test when indicated) for categorical variables. For continuous variables, medians were compared by the Wilcoxon-Mann-Whitney test. Multivariate logistic regression was performed to identify factors independently associated with miscarriage or with *C. trachomatis* infection. Statistical analyses were performed by using Stata version 10.0 (StataCorp LP, College Station, TX, USA).

## Results

Of 395 patients, 9 (2.3%) were excluded because of missing serum or vaginal swab samples. Sociodemographic data for the remaining 386 women are shown in Table 1.

**Table 1:** Characteristics of 386 women, by miscarriage history, in a study of the role of *Chlamydia trachomatis* in miscarriage, University Hospital of Lausanne, Lausanne, Switzerland, November 2006–June 2009*

Characteristic	Control group, no. (%), n = 261	Miscarriage group, no. (%), n = 125	p value
Age, y†			
<35	194 (74.3)	71 (56.8)	0.001
>35	67 (25.7)	54 (43.2)	
No. pregnancies‡			
1	141 (54.0)	38 (30.4)	<0.001
2	78 (29.9)	32 (25.6)	
>2	42 (16.1)	55 (44.0)	
Parity§			
0	160 (61.3)	62 (49.6)	0.066
1	72 (27.6)	41 (32.8)	
>1	29 (11.1)	22 (17.6)	
Origin			
European	217 (83.1)	69 (55.2)	<0.001
Non-European	44 (16.9)	56 (44.8)	
Marital status			
Married	201 (77.0)	90 (72.0)	0.193
Single	49 (18.8)	24 (19.2)	
Divorced	11 (4.2)	11 (8.8)	
Education			
Non–university studies	170 (65.1)	96 (76.8)	0.025
University studies	91 (34.9)	29 (23.2)	
No. lifetime sex partners			
1	58 (22.2)	37 (29.6)	0.031
2 or 3	43 (16.5)	24 (19.2)	
4–6	45 (17.2)	10 (8.0)	
>6	36 (13.8)	10 (8.0)	
No answer	79 (30.3)	44 (35.2)	
Previously used contraceptive method			
Pill	101 (38.7)	36 (28.8)	0.093
Condoms	68 (26.1)	34 (27.2)	
Other	19 7.3)	6 (4.8)	
Never used contraception	73 (28.0)	49 (39.2)	
Smoking status			
Nonsmoker	224 (85.8)	106 (84.8)	0.877
Smoker	37 (14.2)	19 (15.2)	
*C. trachomatis* serologic results			
IgG+	19 (7.3)	19 (15.2)	0.018
IgA+	10 (3.8)	10 (8.0)	0.091
IgG+ and IgA+	7 (2.7)	9 (7.2)	0.037
IgG+ or IgA+	22 (8.4)	20 (16.0)	0.025
*C. trachomatis* PCR			
Cervicovaginal swab	2 (0.8)	5 (4.0)	0.026
Placenta	2 (0.8)	5 (4.0)	0.026
>1 PCR positive	2 (0.8)	6 (4.8)	0.009

*Ig, immunoglobulin. †Age, y, mean + SD: controls, 31.5 + 5.0; women with miscarriage, 33.3 + 6.1; p = 0.002. ‡No. pregnancies, mean + SD: controls, 1.7 + 0.9; women with miscarriage, 2.6 + 0.5; p<0.001. §Parity, mean + SD: controls, 0.5 + 0.8; women with miscarriage, 0.8 + 1.0; p = 0.008.

A total of 16 (4.2%) patients were positive for IgG and IgA against *C. trachomatis*, 22 (5.7%) were positive only for IgG against *C. trachomatis*, and 4 (1.0%) were positive only for IgA against *C. trachomatis*. Prevalence of IgG against *C. trachomatis* was higher in the miscarriage group (15.2%) than in the control group (7.3%; p = 0.018) (Table 1). This association between miscarriage and IgG against *C. trachomatis* remained significant, even after adjustment for age, origin, education, and number of sex partner (odds ratio [OR] 2.3, 95% confidence interval [CI] 1.1–5.1). Similarly, prevalence of IgA against *C. trachomatis* was higher in the miscarriage group (8.0%) than in the control group (3.8%), but this trend was not significant (p = 0.091) by univariate analysis. When adjusted for age, origin, education, and number of sex partners, the association between miscarriage and IgA against *C. trachomatis* was significant (OR 2.7, 95% CI 1.1–7.4).

Multivariate logistic regression including all sociodemographic variables (Table 1) and *C. trachomatis* IgG serologic results identified 5 independent factors positively or negatively associated with miscarriage: *C. trachomatis* IgG–positive serologic results (OR 2.3, 95% CI 1.1–4.9), age >35 years (OR 2.7, 95% CI 1.6–4.4), European origin (OR 0.3, 95% CI 0.2–0.5), marriage (OR 0.4, 95% CI 0.2–0.7), and 1 lifetime sex partner (OR 0.4, 95% CI 0.2–0.7).

*C. trachomatis* DNA was more frequently amplified from products of conception or placenta from women with miscarriage (5 [4.0%] women) than from controls (2 [0.7%], p = 0.026). Most patients with a positive PCR result for placenta also had a positive result for vaginal swab specimens (Table 2). Six of the 7 patients with *C. trachomatis* DNA in the cervicovaginal swab specimen also had positive findings in the placenta. Thus, again, cervicovaginal *C. trachomatis* DNA was more often detected in patients from the miscarriage group (n = 5, 4.0%) than from the control group (n = 2, 0.7%; p = 0.026). All 7 patients with *C. trachomatis* DNA in the cervicovaginal swab also exhibited IgG against *C. trachomatis*, whereas all patients but 1 with *C. trachomatis* DNA in the placenta exhibited IgG against *C. trachomatis* (Table 2). Both patients with *C. trachomatis* DNA and IgG and IgA against *C. trachomatis* belonged to the miscarriage group.

**Table 2:** Clinical history and serologic, PCR, and IHC results of 8 women with samples positive for *Chlamydia trachomatis* by real-time PCR, University Hospital of Lausanne, Lausanne, Switzerland, November 2006–June 2009*

Study group, patient no.	No. pregnancies	Parity	Pregnancy, wk	*C. trachomatis* PCR					Placental histology
				IgG	IgA	Placenta PCR	Vagina PCR	IHC	
Miscarriage group									
235	2	0	8	+	–	+	+	+	Lymphocytes in chorion, acute endometritis
355	1	0	7	+	–	+	+	–	Polymorphonuclear cells in decidua
518	2	0	6	+	–	+	+	+	Subchorial fibrin, lymphocytes in decidua
564	5	2	12	+	+	+	+	+	Lymphocytes in decidua
568	2	1	6	-	–	+	–	–	Lymphocytes in decidua, hemorrhagic necrosis
460	1	0	11	+	+	–	+	+	Presence of eosinophils
Control group									
35	2†	1	37	+	–	+	+	–	Histiocytes, rare calcifications
390	1	1	40	+	–	+	+	+	Chronic deciduitis

*Ig, immunoglobulin; IHC, Immunohistochemical analysis; +, positive; –, negative. †One previous termination of pregnancy.

**Figure 1:**
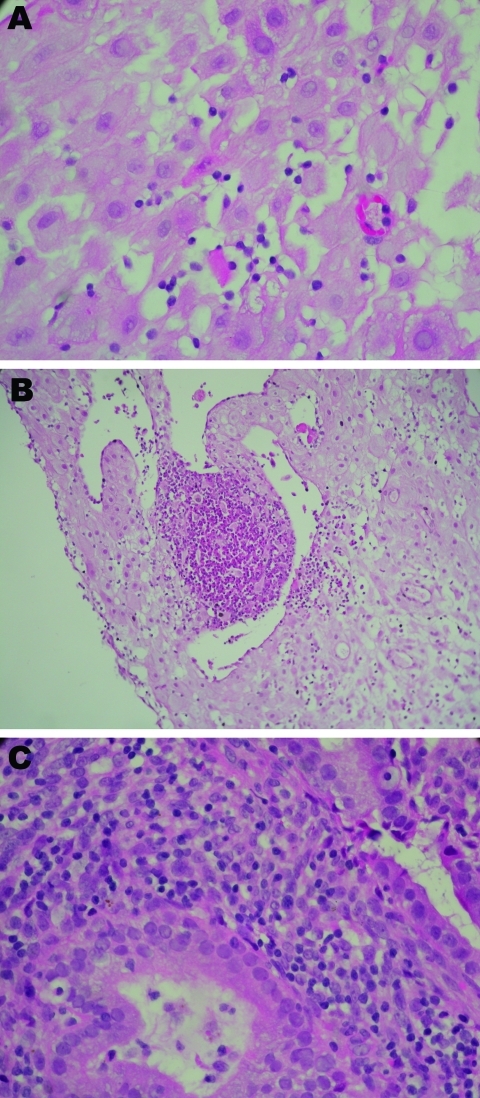
Placental histologic results (*1*) from 3 women with real-time PCR–positive results for *Chlamydia trachomatis* (Table 2). A) Case-patient 390; B) case-patient 235; C) case-patient 564. Histologic analysis shows different degree of periglandular lymphocytes infiltration, with a microabscess in B1. Original magnifications ×600 except B1 (×400).

All placentas were analyzed for inflammation (Figure 1). In the basal plate, inflammatory cells (deciduitis) were present in 15 (39.5%) of 38 patients and 91 (26.1%) of 348 patients with and without *C. trachomatis* IgG–positive serologic results, respectively (p = 0.081). This trend was observed to a lesser extent when *C. trachomatis* IgA serologic results were considered (7 [35.0%] of 20 vs. 99 [26.3%] of 376; p = 0.446).

**Figure 2:**
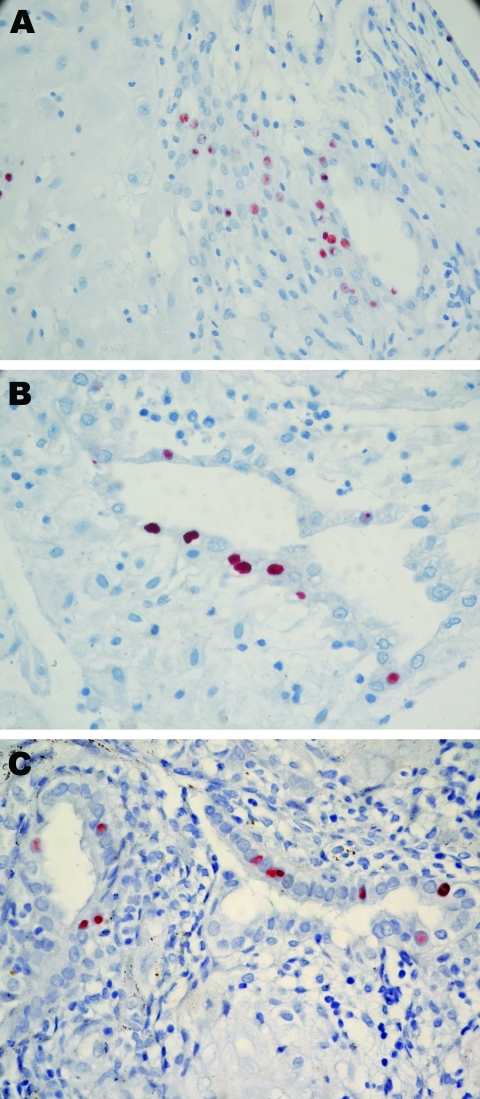
Immunohistochemical analysis of placentas in Figure 1. These placentas were obtained from 3 patients positive for *Chlamydia trachomatis* by real-time PCR. A) case-patient 390; B) case-patient 235; C) case-patient 564. Immunohistochemical analysis demonstrated *C. trachomatis*–infected cells from endometrial glands. Original magnification ×600.

All 8 persons with samples positive for *C. trachomatis* by real-time PCR in the placenta (n = 7) or cervicovaginal swab specimen (n = 7) were tested by IHC (Table 2; Figure 2). *C. trachomatis* was confirmed in 4 of 6 placentas from women with miscarriage and in 1 of 2 placentas from women with uneventful pregnancies, whereas none of the 8 *C. trachomatis* DNA–negative controls randomly selected exhibited the bacteria by IHC. *C. trachomatis* predominantly localized around endometrial glands of the chorion (Figure 2), associated with different degree of inflammation (Figure 1).

We also compared characteristics of patients with (n = 38) and without (n = 348) *C. trachomatis* IgG–positive serologic results. Number of pregnancies, parity, marital status, education, number of lifeltime sex partners, and smoking status were all associated with *C. trachomatis* IgG–positive serologic results by univariate analysis. Women who declined to provide information on the number of sex partners had a *C. trachomatis* IgG prevalence of 12.2%, whereas none of the 95 women who reported having 1 sex partner had *C. trachomatis* IgG–positive serologic result. In multivariate analyses, independent factors positively or negatively associated with *C. trachomatis* IgG–positive serologic results were >2 lifetime sex partners (OR 3.3, 95% CI 1.4–7.7), divorced women (OR 4.9, 95% CI 1.7–14.3), European origin (OR 0.4, 95% CI 0.2–0.9), and attending a university (OR 0.2, 95% CI 0.1–0.6). Age and smoking were not independently associated with *C. trachomatis* IgG–positive serologic results.

## Discussion

We found an association of spontaneous miscarriage with serologic (p = 0.018) and molecular (p = 0.026) evidence of *C. trachomatis* infection. Moreover, *C. trachomatis* in the placenta was documented by specific IHC. *C. trachomatis* was mainly localized in the epithelial cells of endometrial glands.

Several studies have failed to document an association between *C. trachomatis* and spontaneous (*13**–**16*) or recurrent miscarriage (*17**,**18*). However, these studies were conducted >10 years ago, i.e., before the recent dramatic increase in the prevalence and incidence of *C. trachomatis* infection (*1**,**21**,**22*). Because of improved statistical power, such increased prevalence might indicate an association between *C. trachomatis* infection and adverse pregnancy outcomes. Second, sensibility and specificity of diagnostic methods have also improved during the past decade. Thus, the high *C. trachomatis* seroprevalence observed in the control group of several older studies, ranging from 28% to 53% (*16**,**17*) was likely to have resulted from a low specificity of the serologic test used at that time. The *Chlamydia* IgG/IgA kit from R-biopharm we used in the present study exhibited better specificity than did 4 other commercially available tests for detecting IgG against *C. trachomatis* (*23*) and is thus more likely to identify a slight but true association. Moreover, the sensitivity of the *C. trachomatis* TaqMan real-time PCR we used here is high, detecting even <10 DNA copies. This validated assay also detects strains that contain a recently identified 350-bp deletion in the cryptic plasmid (*24**,**25*) because the 71-bp DNA fragment amplified is 93 bp downstream from the deletion (*19*).

The serologic association we observed is unlikely to be due to cross-reactivity with other chlamydial species such as *C. abortus* (previously classified as *C. psittacci* senso lato) because we also observed a molecular association with miscarriage. Moreover, the PCR we used was specific at species level because the *C. abortus* genome contains no cryptic plasmid. Finally, *C. abortus* has been only infrequently associated with miscarriages in humans (*26*), mostly after zoonotic exposure.

Miscarriage could be induced by a persistent asymptomatic *C. trachomatis* infection spreading to the fetal tissue or endometrium. Relatively few miscarriages occur during *C. trachomatis* primary infection, which explains the absence of association with IgA. That several patients exhibited *C. trachomatis*–positive serologic results without *C. trachomatis* DNA suggests that miscarriage might also occasionally be induced by damage from a past chlamydial infection or persistent *C. trachomatis* antibodies that might interfere with embryonic antigens (*2*).

A limitation of our study was the absence of investigation of other infectious etiology of miscarriage. Some viruses can produce chronic or recurrent maternal infection. In particular, cytomegalovirus during pregnancy can reach the placenta by hematogenous spread or by ascending route from the cervix. Parvoviruses also have been implicated in the development of repeated fetal loss. Among bacterial infections, *Ureaplasma urealyticum*, *Mycoplasma hominis*, and bacterial vaginosis have been mostly associated with miscarriages (*27*). In addition, several intracellular bacteria such as *Coxiella burnettii* (*28*), *Brucella abortus* (*29*), and *Waddlia chondrophila* (*11*) have been associated with miscarriage.

Our study shows an association between miscarriage and molecular and serologic evidence of *C. trachomatis* infection. Several previous studies failed to document such an association probably because of the limited number of patients in some of these studies resulting from the lower prevalence of *C. trachomatis* infection in the late 20th century and to lower sensitivity or specificity of diagnostic methods available at that time. The results of our study suggest that all women experiencing a miscarriage should be screened for *C. trachomatis* infection and, if positive, adequately treated to prevent recurrent miscarriages. Moreover, preconceptional screening might be proposed to reduce the prevalence of this adverse pregnancy outcome.
